# Neurosurgical lesions to sensorimotor cortex do not impair action verb processing

**DOI:** 10.1038/s41598-019-57361-3

**Published:** 2020-01-16

**Authors:** Georgette Argiris, Riccardo Budai, Marta Maieron, Tamara Ius, Miran Skrap, Barbara Tomasino

**Affiliations:** 1 Scientific Institute IRCCS “Eugenio Medea”, Polo FVG, San Vito al Tagliamento, PN Italy; 2 Unità Operativa di Neurologia, Azienda Sanitaria Universitaria Integrata S. Maria della Misericordia, Udine, Italy; 3 Fisica Medica, Azienda Sanitaria Universitaria Integrata S. Maria della Misericordia, Udine, Italy; 4 Unità Operativa di Neurochirurgia, Azienda Sanitaria Universitaria Integrata S. Maria della Misericordia, Udine, Italy; 5 Present Address: Columbia University Medical Center, Neurological Institute, 710 West 168th Street, New York, NY 10032 USA

**Keywords:** Language, Motor cortex, Neurophysiology, Human behaviour

## Abstract

There is ongoing debate regarding the role that sensorimotor regions play in conceptual processing, with embodied theories supporting their direct involvement in processing verbs describing body part movements. Patient lesion studies examining a causal role for sensorimotor activation in conceptual task performance have suffered the caveat of lesions being largely diffuse and extensive beyond sensorimotor cortices. The current study addresses this limitation in reporting on 20 pre-operative neurosurgical patients with focal lesion to the pre- and post-central area corresponding to somatotopic representations. Patients were presented with a battery of neuropsychological tests and experimental tasks tapping into motor imagery and verbal conceptual verb processing in addition to neurophysiological measures including DTI, fMRI, and MEP being measured. Results indicated that left tumor patients who presented with a lesion at or near somatotopic hand representations performed significantly worse on the mental rotation hand task and that performance correlated with MEP amplitudes in the upper limb motor region. Furthermore, performance on tasks of verbal processing was within the normal range. Taken together, while our results evidence the involvement of the motor system in motor imagery processes, they do not support the embodied view that sensorimotor regions are necessary to tasks of action verb processing.

## Introduction

According to theories of embodied cognition, the representation of concepts activates the same sensorimotor modalities that are responsible for perceiving and acting upon those concepts^1–3^. In the strictest sense of embodiment, language comprehension is achieved through *simulation*- that is, the same sensorimotor neural circuitry that is involved when interacting with an object through direct experience is *necessarily* reactivated for comprehending that object^4,5^. At the other extreme, unembodied theories contend that concepts are stored and accessed in modality-independent systems distinct from sensory processing; sensorimotor subsystems may only be indirectly activated as a result of post-conceptual processing^6,7^. These theories have been proposed along a continuum^8^, each positing the direct/indirect involvemment of sensorimotor systems in conceptual processing to varying extents. The fundamental question remains as to whether or not sensorimotor regions are necessary to conceptual processing.

Evidence in favor of motor processing in linguistic comprehension has come from research on the processing of action verbs and motor cortical activation. Functional magnetic imaging (fMRI) studies have demonstrated that the processing of action words has led to somatotopic activation in regions that are associated with the specific body part involved in the action execution of that word (e.g., to “chew”, to “grasp”, to “kick” activate areas close to motor representations for the face, arm, and leg, respectively)^9–11^. In a similar vein, the reverse link from motor to language processing has also been shown, with transcranial magnetic stimulation (TMS)-induced facilitation of processing arm- and leg-related words when stimulation was applied to arm- and leg-related motor and premotor areas, respectively^12^. Furthermore, recordings of motor-evoked potentials (MPEs) have demonstrated an increase in amplitude in hand and foot muscular activation when listening to sentences describing hand or foot actions, respectively^13^.

Criticisms to embodiment contend that sensorimotor activation may reflect a cascade of processing from other regions where lexico-semantic processing effectively occurs^14^. For instance, Mahon and Caramazza have argued that phonological processing of a word can even precede lexical node selection^14^, signaling a spreading of activation not limited to discrete levels^15^. Likewise, they claim that such spreading is responsible for automatic activation of the motor system when naming tools like *hammer*. However, challenging this criticism, TMS studies have demonstrated either an interference or facilitation of conceptual processing related to action words when targeting motor regions^12,16^. Furthermore, it has been demonstrated that the critical window for lexical processing and the window of interest for modality-specific (e.g., motor) processing both occur around 200 ms post-target onset^17,18^. While other criticisms have pointed out that embodiment theories rely on inferential thinking and do not imply causation^19^, TMS and electrophysiological measures have provided evidence to the contrary.

Patient studies have also offered critical insight into the involvement of motor regions in action-related stimuli. The role of the motor system to action verb processing has been evidenced in patients with motor neuron disease^20^, Parkinson’s disease^21^, and stroke^22^. However, in such patient studies, brain deficit has not always been strictly limited to motor processing regions and have represented a more global deficit as dictated by the nature of the disease. Although evidence has been provided towards somatotopic activation of motor regions associated with verbs related to specific body parts^23^, patient lesions were not restricted to the sensorimotor cortex, confounding the interpretability of findings. Tomasino and colleagues instead tested motor activation in action word processing in a group of neurosurgical patients with selective lesions to the sensorimotor cortex and found that the creation of mental images for verbs describing actions was slower for actions that involved a body part whose somatotopic representation fell within the lesion site^24^. Such studies attest to the importance of considering action representations in patients with focalized lesions in order to draw more solid conclusions as to sensorimotor involvement.

In addition to lexico-semantic tasks, the involvement of motor and somatosensory cortices in tasks of motor imagery- mentally simulated movements that do not lead to overt motor production^25^- has also been explored. The mental rotation of body parts, for instance, has been used to investigate whether or not motor regions are automatically or strategically engaged in solving task demands. Modulation of reaction time in mental rotation is typically explained by biomechanical constraints of body movements in the act of motor simulation^26^. Using single-pulse TMS, Tomasino and colleagues demonstrated that stimulation to the left motor hand area selectively increased reaction times for mental rotation of hands but not for letters^27^. Extended to the patient domain, Tomasino and colleagues also reported an interference effect in tasks involving motor imagery in a patient receiving direct electrical current to the motor cortex (M1) to relieve chronic pain^28^. However, not all studies have converged on this finding. Sauner and colleagues found no effect of M1 stimulation on handedness judgments in a mental rotation task^29^ whereas others have found an effect only when explicitly requiring imagined motor execution^30^ or retrieving motor information in a semantic task^31^. Discrepant findings have thus been interpreted to be the result of the stimuli used and the strategy invoked by the experimental design^32^. These results reflect a flexibility of processing due to an indirect connection between the motor and language systems via sensorimotor representations^24^. Patient studies offer the possibility to discern whether the involvement of motor regions is necessary to lexico-semantic processing.

In the current paper, we investigate whether sensorimotor regions play a causal role in influencing performance in tasks that presumably engage sensorimotor activation. We tested twenty patients with brain glioma (14 left and 6 right) to the pre- and post-central gyrus at or around sites corresponding to somatotopic representations of body parts (i.e., hand, foot, or mouth) on a battery of neuropsychological tests that engage mental rotation, action verb processing and motor imagery. Additionally, we recorded neurophysiological measurements of fMRI mean signal during hands, feet and mouth movements, white matter integrity using diffusion tensor imaging (DTI) and motor evoked potentials.

## Materials and Methods

### Participants

**Figure 1:**
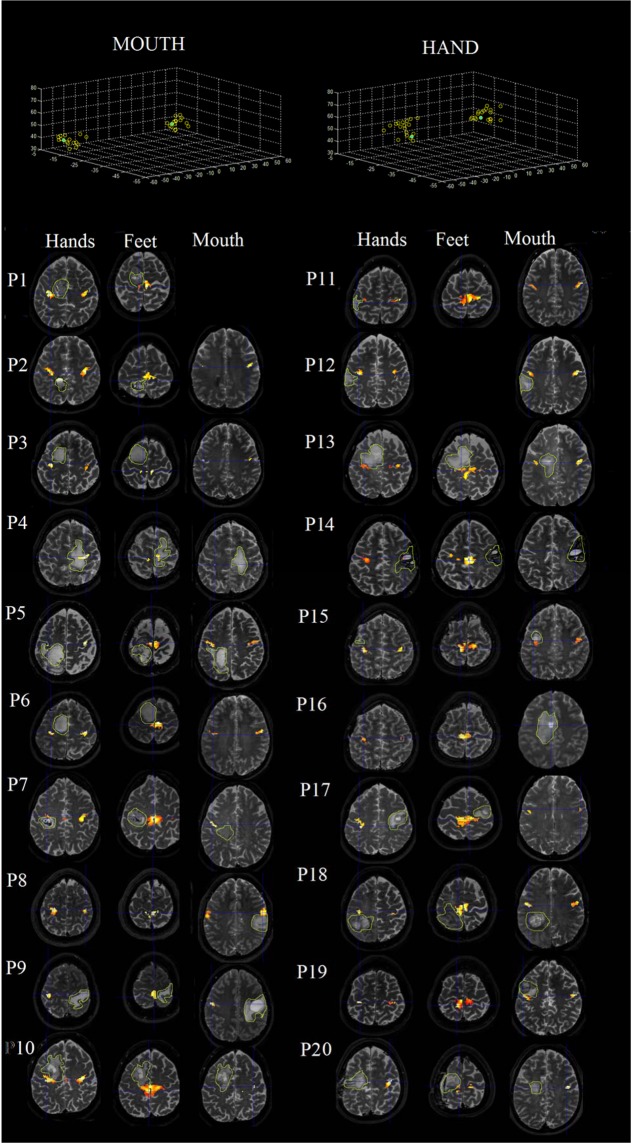
Upper: MNI coordinates of peak activations for mouth and hand region. Yellow circles indicate patient activations and green dots indicate mean control activations. As can be observed, peak activations between patients and controls were similar, indicating that somatotopic organization was preserved despite lesion. Lower: Axial view of the localization of patients’ lesions with somatotopic activations divided by body part (images are presented in standard left-right view). The green line demarcates the tumor site.

Twenty neurosurgical patients were included in the present study for having a glioma (13 high grade glioma (HGG) and 7 low grade glioma (LGG)) involving the pre- and post-central area (see Fig. 1 for motor-related fMRI maps): fourteen right-handed (8 females, 6 males) patients (mean age: 49.5 ± 16.65, range: 16–70; mean education: 13.14 ± 2.6) with focal glioma in the left hemisphere (LH) and six right-handed (5 males, 1 female) patients (mean age: 37.33 ± 7.84, range: 26–48; mean education: 13 ± 4.47) with focal glioma in the right hemisphere (RH; see Table 1 for glioma type). The Edinburgh Inventory test^33^ was used to assess handedness. All patients (except P19) presented with a sensory or motor disturbance to the arm or the leg that was contralateral (see Table 1 for a list of patients’ clinical details). In the case of P19, the presence of the tumor was early identified due to a loss of consciousness that preceded potential motor or sensory deficits. T1-weighted MR imaging revealed lesions measuring 51.29 ± 35 cc for LH patients (volume range: 4.8–113.6cc) and 60.07 ± 45.09cc for RH patients (range 2.7–128.4; see Table 1 for individual volumes) (*P* > 0.05, n.s.). All patients participated in the study one week before surgery. Twenty right-handed healthy controls (10M, 10F; mean age: 41.9 ± 13.39, mean education: 12.75 ± 3.02, were involved in the study.

**Table 1:** Clinical details of all twenty patients included in the study.

Pat_ID	Sex	Age	Education	Tumor_Hemi	Tumor_grade	Tumor_Loc	Reported dysfunction	Tumor_Vol (mm^3^)
1	F	67	13	Left	HGG	Premotor	Deficit in arm movement (right)	36.1
2	F	49	13	Left	LGG	Motor	Deficit in foot movement (right)	19.4
3	M	39	16	Left	LGG	Premotor	loss of consciousness	29.5
5	M	70	13	Left	HGG	Sensorimotor	tingling in arm and leg (right)	113.6
6	M	24	17	Left	LGG	Motor	stiffening of leg (right); loss of consciousness	37.2
7	F	65	13	Left	HGG	Motor	Tingling in fingers (right)	43.7
10	M	70	13	Left	HGG	Motor	Clonic seizures arm (right); loss of consciousness	73.4
12	M	46	13	Left	LGG	Sensorimotor	Deficit in hand strength (right); expressive aphasia before loss of consciousness	45.5
13	F	42	18	Left	LGG	Premotor	Cloned hemibody sensation (right)	104.7
15	F	16	10	Left	HGG	Premotor	Loss of consciousness	4.8
16	F	38	13	Left	HGG	Parasaggital	Clonic seizures arm and reduced foot strength (right)	104.9
18	M	57	11	Left	HGG	Sensorimotor	Tingling in hand (right); loss of consciousness;	29.8
19	F	58	8	Left	HGG	Motor	Loss of consciousness	18.3
20	F	52	13	Left	HGG	Motor	Deficit in strength of hemisome (right)	57.1
4	M	44	8	Right	HGG	Motor	loss of sensitivity in arm and foot (left)	40.8
8	M	33	18	Right	LGG	Premotor	Tingling in arm (left); loss of consciousness	45.5
9	M	37	18	Right	HGG	Motor/Premotor	Loss of sensitivity in hemisome (left)	97.6
11	M	26	13	Right	LGG	Motor	Clonic seizures (left);Loss of consciousness	2.7
14	M	36	13	Right	HGG	Sensorimotor	Tingling in hand and mouth(left)	128.4
17	F	48	8	Right	HGG	Motor	Deficit in hand strength (left)	45.6
	9 F/11 M	45.8 ± 15.4	13.1 ± 3.1	14 L/6 R	13HGG/7LGG	—	—	53.9 ± 37.2

HGG = high grade glioma; LGG = low grade glioma.

The study was approved by the Ethics Committee of the IRCCS Eugenio Medea” Scientific Institute and carried out in accordance with the 2013 Fortaleza version of the Helsinki Declaration and subsequent amendments. All participants provided informed written consent prior to partaking in the study.

### Patient Self-Report and Neurological Motor Examination

Consistent with lesion location (see Fig. 1), we ascertained that the lesion involved the sensorimotor network, as patients reported motor disturbances, aberrant or loss of sensation to limbs, and/or muscular weakness (see Table 1). Furthermore, 40% of patients reported having experienced an episode of consciousness loss that was seizure-induced.

### Lesion localization

Contrast-enhanced T1- and T2-weighted MRI scans were performed 7–10 days prior to surgery in order to determine the preoperative tumor location. DTI and anatomical and functional images were acquired from both patients and healthy controls using a Philips Achieva 3-T (Best, Netherlands) scanner. A SENSE-Head-8 channel head coil and a custom-built head restrainer were used to minimize head movements.

As brain shift compensation may occur post tumor excision, preoperative localization was the preferred method for comparison with somatotopic activations related to mouth/hand/foot representations.

### Imaging data acquisition and analysis

#### fMRI data acquisition

Functional images were obtained using a single-shot gradient echo echoplanar imaging (EPI) sequence [34 axial slices, TR = 2500 ms, TE = 35 ms, FOV = 230.000 mm, matrix: 128 × 128; slice thickness of 3 mm with no gaps, 90° flip angle, voxel size: 1.79 × 1.79 × 3.3 mm, 4 dummy images to allow the magnetization of the spins to stabilize to a steady state]. 3D images were acquired using a T1-weighted 3D magnetization-prepared, rapid acquisition gradient-echo fast field echo (T1W_3D_TFE SENSE) pulse sequence (TR = 8.1007 ms, TE = 3.707 ms, FOV = 240.000 mm, 190 sagittal slices of 1 mm thickness, flip angle = 8°, voxel size: 1 × 1 × 1] and T2-weighted 3D magnetization-prepared, rapid acquisition gradient-echo fast field echo (T2W_3D_TFE SENSE) pulse sequence (TR = 2500 ms, TE = 368.328 ms, FOV = 240.000 mm, 190 sagittal slices of 1 mm thickness, flip angle = 90°, voxel size: 1 × 1 × 1). Presentation^®^ software (Version 9.9, Neurobehavioral Systems Inc., CA, USA) was used for stimuli presentation and synchronization with the MR scanner. Participants viewed the stimuli via a VisuaStim Goggles system (Resonance Technology). Participants practiced the tasks outside of the scanner prior to in-scanner registration.

#### fMRI data analysis

Statistical analysis was performed using MATLAB r2007b (The Mathworks Inc., Natick, MA/USA) and SPM12 (Statistical Parametric Mapping software, SPM; Wellcome Department of Imaging Neuroscience, London, UK; http://www.fil.ion.ucl.ac.uk/spm). In brief, pre-processing included: spatial realignment, segmentation producing the parameter file used for normalization, re-sampling to a voxel size of 2 × 2 × 2 mm, and spatial smoothing with a 6 mm FWHM Gaussian kernel. Data was statistically analyzed using a general linear model (GLM), which included six nuisance regressors for head movement. Blocks were convolved with a standard two-gamma hemodynamic response function^34^. A significant threshold of p < 0.05 was used, which was FWE-corrected for multiple comparisons at the cluster level, utilizing a height threshold of p < 0.001, uncorrected at the voxel level.

#### Motor localizer

All patients and healthy controls were asked to perform motor localizer tasks, which were used for somatotopic mapping of the cortex and consisted of mouth, hand, and feet movements. For patients, movements of each body part were performed across three different runs in block design, each run consisting of four blocks (15 seconds each) dedicated to the movement of each body part (bilaterally). Activation was defined as movement versus rest for each body part. Upon hearing “Move”, patients were instructed to perform i) lip protrusion, ii) left and right hand movements (open/close) and iii) left and right foot movements (moving the top of the foot towards their head). In the case of healthy controls, only two runs were performed, with only mouth and hand regions being localized.

Region of interest (ROI) analysis. We performed an ROI analysis on the activations from the motor task. Using the SPM Anatomy toolbox (http://www.fz-juelich.de/inm/inm-1/EN/Forschung/_docs/SPMAnatomyToolbox/SPMAnatomyToolbox_node.html), we derived the cytoarchitectonically-defined multi-parameter maps (MPMs) of the primary motor cortex (M1; Brodmann area 4). Utilizing the WFU_Pickatlas (https://www.nitrc.org/projects/wfu_pickatlas/), ROIs were defined for each subject as all contiguous voxels displaying significantly greater activation when performing clenching hand, mouth, and foot movements as compared to baseline (threshold of *p* < 0.05, FWE-corrected). ROIs were further constrained to encompassing only those active voxels that were located within the MPMs of M1. Motor maps were constructed from average activations across runs, for each movement type, and, in the case of patients, these action-execution-related activations were outlined with respect to the tumor site (see Fig. 1 for peak activation plots and Supplementary (S2) Table 1 for peak coordinates). For healthy controls, ROIs were generated as a result of the second-level group analysis according to the same cytoarchitectonically-defined procedure above (see Fig. 1 for maps). We further extracted the number of active voxels and beta-values used to generate single mean parameter estimates for each ROI.

Peak activation analysis. Peak MNI coordinates for patients and controls were compared to ensure that clusters did not relocate due to the effect of lesion. To achieve this, we first calculated the mean XYZ coordinates for the control group to serve as an index of normality. Next, we calculated the Euclidean distance between each participant (both patients and controls) and the mean XYZ of the control group. We then performed an independent-samples t-test to see if the Euclidean distance from the mean differed between patients and controls.

#### Diffusion Tensor Imaging (DTI)

DTI data acquisition. Whole-brain diffusion tensor data were acquired using an axial diffusion-weighted, single-shot, spin-echo planar imaging sequence (repetition time = 8880 ms; echo time = 70 ms, bandwidth = 3135 Hz/pixel; flip angle = 90°; FOV = 240 × 240 cm; 57 contiguous axial slices, 2.1 mm slice thickness; matrix size = 128 × 128 voxels). Two *b*-values were used; seven images at 0 s/mm^2^ (no diffusion weighting) and 64 non-coplanar images at 1000 s/mm^2^ (diffusion-weighting *b*-value) were acquired. The derived gradient directions were uniformly distributed on a sphere.

DTI data processing: Corticospinal tract fibers reconstruction. DTI images were analyzed in DTIStudio (version 3.0.3) according to the following steps: realignment using the AIR program^35^ and inspected for motion-related or technically induced artifacts; multivariate linear fitting of the 64 elements of the diffusion tensor at each voxel; tensor diagonalization by matrix rotation to obtain the three eigenvalues and eigenvectors that were used to derive the fractional anisotropy (FA) maps. Fiber orientation was defined as the eigenvector with the corresponding largest eigenvalue. Fiber tracking was performed using the fiber assignment by continuous tracking (FACT) method^36^ (FA threshold >0.2; angle threshold = 45°). Wanaka and colleagues’ multi-ROI approach was used to reconstruct the corticospinal tract^37^. ROIs were delineated on non-diffusion weighted b0 images from the first DTI acquisition and subsequently mapped to the FA image.

### Motor-Evoked potentials (MEPs)

MEPs were induced by MagPro stimulator (MagVenture) and data acquired using the Dantec Keypoint multi(6)-channel electromyography system. Cortical magnetic stimulation was bilaterally applied to the motor cortex and the elicited action potentials were recorded via a surface electrode placed at the first dorsal interosseous muscle of either hand. The response was measured as both the time (milliseconds) required for the electrical impulse to travel from the stimulation site to an electrode placed at the recording site and the amplitude (millivolts) of the evoked response.

### Neuropsychological testing

Patients were also presented with a battery of neuropsychological tests assessing different aspects of cognition. Overall, they succeeded performing the tasks (see Supplementary Material S2. Table 1). Whenever appropriate, all scores were corrected for age and education. All patients were given the Raven’s Coloured Progressive Matrices test^38^ as a measure of nonverbal intelligence, with all performing well (range: 26–34.5; cutoff: 18). A different subset of tests was used to assess function in left versus right hemisphere tumor patients, as domains of impairment have been shown to differ by hemisphere^39^. As a large body of research has supported a right lateralization of spatial working memory and visuospatial attention^40–43^, right tumor patients were presented with Corsi block span (both forward and backward)^44^ as a measure of visuo-spatial short term memory (STM). In forward span, only one patient performed slightly below normal (range: 3.54–5.92; cutoff: 3.75), whereas in backward span, which purportedly engages the central executive to a greater extent, all patients apart from one performed below the cutoff (range: 3.24–5.31; cutoff: 3.75), potentially suggesting a slight general impairment in working memory processing. Right tumor patients were also tested for constructional apraxia (i.e., Figure drawing)^45^, visualspatial/constructive ability and planning (Clock-drawing test (CDT))^46^ attentional neglect (Behavioral Inattention Test (BIT))^47^ and visual-conceptual and visuomotor tracking (Trailmaking Test (TMT))^48^ and demonstrated performance well within normal range apart from one patient performing below the cutoff on the CDT.

Alternatively, as left hemisphere tumors have been more typically associated with deficits in language processing and praxis^49^, tests administered to left tumor patients included verbal STM (digit span, both forward and backward)^50^, buccofacial^45^, and ideomotor apraxia^51^, in addition to language comprehension (Token test)^52^, noun naming (subsection of the BADA)^53^ and phonological fluency^54^. Patients again generally performed within the normal range on these tests, except one patient performing below the normal range on verbal STM backward, ideomotor apraxia and verbal fluency, and two patients performing slightly below the cut off on noun naming, and another three on verbal fluency (see Supplementary Material S2. Table 1 for patient task performance).

### Experimental tasks

For all tasks, stimuli were presented on a white background of a 19” LCD monitor by Presentation software (Neurobehavioral Systems Inc., CA/USA, version 9.90). Subjects were seated approximately 50 cm from a monitor with 48 cm diagonal and were instructed to keep their hands and feet as still and relaxed as possible. Subjects responded by mouse press. Responses were coded as correct (1 point) or incorrect (0 points). For a complete description of experimental tasks, see the Supplementary Material Experimental Tasks section. The following abilities were assessed:

- *General motor imagery ability*- this was assessed using an adapted Italian computerized version of the Florida Praxis Imagery Questionnaire (FPIQ)^55^, in which participants were presented with action scenarios tapping into different aspects of learned skilled movement. Participants were required to answer imagery questions about joint movement or the spatial position of the hands during action production− e.g., Imagine you are using a handsaw. Which joint moves more, your shoulder or your wrist?
- *General motor imagery ability-* this was assessed by the presentation of a mental rotation task in which participants were presented with rotated images of hands and feet and had to decide if the hand or foot represents the right or left.
- *Conceptual knowledge of actions-* this was assessed using a written verb subtest of the Kissing and Dancing Test (KDT)^56^ adapted for Italian speakers. Participants were presented with a probe word and had to decide which of two choice verbs corresponded to it. For example, if the probe word was “Writing”, the correct target word was “Typing” and the distractor word was “Stirring”.
- *Lexical grammar processing-* this was assessed using a subset of Italian verbs relating to the hand, face, and foot previously presented by Tomasino and colleagues^57^ and originally taken from the Italian corpus of Laudanna and colleagues^58^. In a previous study, participants were asked to silently read and decide on the syntactic subject of action-related and nonaction-related verbs, with TMS-induced MEPs increases for first-person but neither for third-person action verbs nor first- or third-person nonaction verbs^59^. Following that study, we presented participants with verbs in the first- or third-person singular or plural and asked them to decide if the word represents a third-person singular form.
- *Verb naming*- this was assessed using the verb oral naming task from the Batteria per L’Analisi dei Deficit Afasici (BADA)^53^. Participants were presented with a series of pictures and asked to name the corresponding verb associated with it.

### Statistical analysis

Data were analyzed using the R system for statistical computing (Ver. 2.8.1)^60^. Missing data was coded as *nan* and not included in the analysis. P-values were computed adopting the Satterthwaite approximation for degrees of freedom^61^. Z-scores were calculated for patient task performance based on the mean and standard deviation of control group performance, with a one-tailed significance cutoff of ±1.64. In the case of unequal sample size between groups, Welch’s t-test was performed; otherwise, student’s t-test was used. Correlations were performed under the assumption of normality and two-tailed significance assessed. Mean and standard deviations of performance are reported as accuracies scaled between 0 and 1. In the case of multiple patients demonstrating Z-scores significantly outside of the range of controls on a given task, only the maximum Z-score (when patient activations/performance are lower with respect to controls) or minimum Z-score (when activations/performance are higher) is reported.

## Results

### Neurophysiological measures

All patient peak MNI coordinates, active voxel counts and mean parameter estimates (MPE) were statistically compared to control performance by Z-score comparison. As we do not have control data for the foot region, only patient hand and mouth activations are reported for significance.

#### Peak MNI coordinates, active voxel count, and fMRI Mean Parameter Estimates (MPE)

Peak MNI coordinates for patients and controls were compared and confirmed homogeneity between groups such that any relationship with performance could not be attributable to gross anatomical differences in somatotopic organization (P-value range: 0.073–0.633).

In contrast, the analysis of active voxel count and MPEs revealed that the lesion impacted sensorimotor representations in terms of a reduction in dimension of activation (i.e., number of active voxels) and functionality (i.e., strength of MPE activation) for patients relative to controls.

Peak MNI coordinates. There were no appreciable group differences; no patient demonstrated peak coordinates outside of the normal control range (see Supplementary Material Table 2) in more than one axis (for a plot of coordinates, see Fig. 1).

Active voxel count. Overall, a considerable number of patients demonstrated an active voxel count significantly outside of the range of controls, indicating that their motor representation was reduced due to the lesion.

Hand Localizer. Three of the 14 LH patients (P5, P7, and P20) demonstrated a significant decrease in the number of active voxels in the left hemisphere (maximum: *Z-*score = −3.61, p < 0.001), with P5 and P20 also demonstrating a significant reduction in the right hemisphere (maximum: *Z-*score = −2.09, p = 0.018). Three other patients displayed right hemisphere voxel counts outside of the normal range (1 lower: P16 (*Z*-score = −2.33, p = 0.01); 2 higher: P18 and P19 (minimum: *Z*-score = 2.04, p = 0.02).

Three out of the 6 RH patients demonstrated a significant decrease in voxel count in the left hemisphere (P4, P8, and P9; maximum: *Z-*score = −1.78, p = 0.04), with P8 and P9 also displaying a significant decrease in the right hemisphere (maximum: *Z-*score = −2.26, p = 0.012). Two other patients (P14 and P17) also demonstrated a voxel count decrease in the right hemisphere (maximum: *Z-*score = −3.26, p < 0.001).

Mouth Localizer. Six of the 14 LH patients (P2, P3, P6, P10, P16, and P20) demonstrated significantly reduced voxel count (maximum: *Z*-score = −2.02, p = 0.02), and 1 LH patient (P5) an increase in the left hemisphere (*Z*-score = 2.37, p = 0.009). In the right hemisphere, 4 LH patients (P7, P10, P16, and P20) demonstrated a reduction in voxel count (maximum: *Z-*score = −2.19, p = 0.014) whereas 2 LH patients (P5 and P15) demonstrated a significant increase in voxel count (minimum: *Z-*score = 1.81, p = 0.035. Notably, P5 presented with a bilateral decrease in voxel count for the hand localizer though a bilateral increase in voxel count for the mouth localizer.

Conversely, all of the RH patients presenting with voxel counts significantly outside of the range of the control group demonstrated a relative decrease in voxel count. Three out of the 6 RH patients (P4, P8, and P9) demonstrated a significantly lower voxel count in the left hemisphere (maximum: *Z-*score = −1.78, p = 0.04) and 5 out of the 6 RH patients (P4, P8, P9, P14, and P17) demonstrated a significantly lower voxel count in the right hemisphere (maximum: *Z-*score = −2.25, p = 0.012).

**Figure 2:**
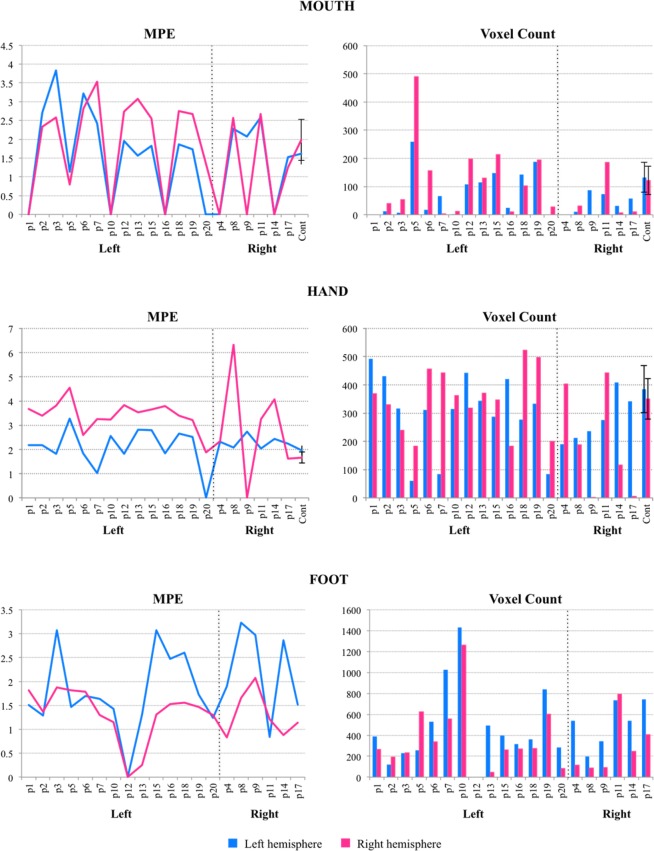
Mean Parameter Estimates (MPE) and voxel count of activation associated with each somatotopic region. Patients are divided by lesion hemisphere (Left or Right), with a dashed line indicating this division. Legend refers to the hemisphere of analysis. Note: P1 did not perform the mouth localizer and P12 did not complete the foot localizer.

Mean parameter estimates (MPE). Mean parameter estimates are plotted in Fig. 2. Overall, a considerable number of patients demonstrated peak activation significantly outside of the outside the normal range, indicating that their motor representation was reduced due to the lesion.

Hand localizer. Eight out of the 14 LH patients demonstrated peak activation in the left hemisphere outside the normal range- 6 higher than controls (P5, P10, P13, P15, P18, and P19; minimum: *Z-*score = 2.77, p = 0.003) and 2 lower (P7 and P20; maximum: *Z-*score = −4.67, p < 0.001; see Table 2). Furthermore, 6 of these 8 had tumors closest to the hand region and the other 2 had tumors closest to the mouth region. Notably, all but one LH patient (P20) also demonstrated peak activation in the right hemisphere that was significantly higher than controls (minimum: *Z-*score = 4.07, p < 0.001).

**Table 2:** Patients’ Mean Parameter Estimates (MPE) and number of active voxels from localizer.

P_ID	Les_Hem	H_LH_MPE	H_RH_MPE	F_LH_MPE	F_RH_MPE	M_LH_MPE	M_RH_MPE	H_LH_vox	H_RH_vox	F_LH_vox	F_RH_vox	M_LH_vox	M_RH_vox
1	LH	2.17	**3.67**	1.51	1.82	ne	ne	492	369	390	267	ne	ne
2	LH	2.19	**3.39**	1.29	1.37	**2.71**	2.33	431	330	121	193	**13**	40
3	LH	1.81	**3.81**	3.07	1.88	**3.83**	2.58	316	239	228	232	**8**	54
5	LH	**3.28**	**4.55**	1.47	1.81	**1.13**	**0.79**	**61**	**183**	258	627	**259**	**489**
6	LH	1.85	**2.60**	1.70	1.78	**3.22**	2.82	311	457	528	339	**18**	156
7	LH	**1.03**	**3.26**	1.64	1.29	**2.42**	**3.53**	**85**	443	1026	556	66	**4**
10	LH	**2.57**	**3.24**	1.43	1.14	**0.00**	**0.00**	314	362	1431	1265	**1**	**12**
12	LH	1.83	**3.83**	ne	ne	1.96	2.73	442	318	ne	ne	108	198
13	LH	**2.82**	**3.53**	1.31	0.25	1.56	**3.07**	344	371	494	48	115	131
15	LH	**2.79**	**3.65**	3.07	1.31	1.83	2.55	287	348	400	263	147	**213**
16	LH	1.85	**3.80**	2.47	1.53	**0.00**	**0.00**	421	**184**	315	271	**25**	**10**
18	LH	**2.65**	**3.39**	2.61	1.56	1.87	2.74	277	**523**	360	273	142	102
19	LH	**2.51**	**3.22**	1.72	1.47	1.74	2.67	334	**497**	839	601	187	195
20	LH	**0.00**	1.89	1.24	1.30	**0.00**	1.37	**84**	**201**	286	84	**0**	**28**
4	RH	**2.31**	**2.33**	1.89	0.83	**0.00**	**0.00**	**190**	404	538	114	**0**	**0**
8	RH	**2.07**	**6.32**	3.23	1.65	**2.28**	2.57	**213**	**189**	199	87	**10**	**32**
9	RH	**2.74**	**0.00**	2.97	2.08	**2.07**	0.00	**237**	**1**	342	93	87	**0**
11	RH	**2.04**	**3.26**	0.84	1.21	**2.59**	2.67	275	442	736	792	74	186
14	RH	**2.44**	**4.07**	2.86	0.88	**0.00**	**0.00**	409	**117**	539	247	**32**	**7**
17	RH	**2.24**	**1.61**	1.51	1.14	1.52	1.25	342	**5**	742	405	57	**11**
Cont__M	—	*1.96*	*1.66*	*ne*	*ne*	*1.62*	*1.98*	*357.9*	*350.7*	*ne*	*ne*	*132.71*	*122.00*
Cont_SD	—	*0.20*	*0.23*	*ne*	*ne*	*0.29*	*0.55*	*83.18*	*71.65*	*ne*	*ne*	*53.29*	*50.14*

Performance that significantly differed from controls, as assessed by Z-score comparison, is in bold face; ne = task not executed;

Cont_M = control group mean; Cont_SD = control group standard deviation; LH = left hemisphere; RH = right hemisphere;

MPE = mean parameter estimate; vox = number of active voxels; H = Hand; F = Foot; M = Mouth.

A difference between left and right hemisphere MPEs (LH_MPE **−** RH_MPE) was calculated for both the LH patients and control group to see if there was a disproportionate difference in intensity of activation across hemispheres potentially due to tumor interference or compensatory activation. Indeed, there was a significant difference between groups [F(1, 28) = 107.15, p < 0.001, *d* = 3.2], with LH patients demonstrating a negative difference (LH group: *M* = −1.32, *SD* = 0.59; controls: *M* = 0.29; *SD* = 0.19); activation was significantly higher in the right hemisphere, displaying an *enhancement* of activation as compared to controls, confirmed by a lack of significant difference in left hemisphere peak activations alone [F(1, 28) = 0.42, p = 0.52]. Furthermore, when controlling for the hemispheric difference in active voxels (number of active voxels (LH) **−** number of active voxels (RH)) by entering it as a covariate in the linear regression, the between-group change in peak intensity across hemispheres remained significant [F(2, 27) = 111.15, p < 0.001, η^2^ = 0.8], indicating an increase in right hemisphere peak activation that is not attributable simply to an increase in size of localizer activation.

All 6 RH patients demonstrated peak activation in the left hemisphere that was outside the range of normal- 4 higher than controls (P4, P8, P11, and P14; minimum: *Z*-score = 1.85, p = 0.032) and 2 lower (P9, and P17; maximum: *Z*-score = −1.74, p = 0.04). Furthermore, 5 of these patients had tumors located closest to the hand region. Interestingly, all but one RH patient (P11) also demonstrated peak activations in the right hemisphere that were significantly higher than that of controls (minimum: *Z*-score = 1.79, p = 0.037), even though they presented with right hemisphere tumors.

Mouth localizer. Of the 13 out of 14 LH patients from which localizer activations were recorded, 7 LH patients demonstrated a peak activation that was either above or below that of controls- 4 higher than controls (P2, P3, P6, and P7; minimum: *Z*-score = 2.75, p = 0.003) and 3 lower (P5, P16, and P20; maximum: *Z*-score = −1.68, p = 0.047; see Table 2). Furthermore, 4 of these patients had a tumor closest to the mouth region. Five out of 14 patients also demonstrated peak activations either significantly above or below controls in the right hemisphere- 2 higher than controls (P7 and P13; minimum: *Z*-score = 1.98, p = 0.024) and 3 lower (P5, P10, and P16; maximum: *Z*-score = −2.16, p = 0.015). Furthermore, 4 out of 5 had tumors located closest to the mouth region. A difference between left and right hemisphere MPEs (LH_MPE**−**RH_MPE) for the mouth region was calculated for both RH and control groups but a significant difference did not emerge [F(1, 28) = 0.002, p = 0.96].

Of the 6 RH patients, 5 RH patients demonstrated peak activation in the left hemisphere that was either above or below that of controls, with 2 higher (P8 and P11; minimum: *Z*-score = 3.27, p < 0.001) and 3 lower (P4, P9, and P14; *Z*-score = −5.59, p < 0.001). Furthermore, 3 of these patients had a tumor closest to the mouth region. Only P4 and P14 also demonstrated peak activations in the right hemisphere that were significantly below that of controls (*Z*-score = −3.36, p < 0.001), these two patients demonstrating a reduction in peak activation in both hemispheres and both presenting with tumors in the mouth region.

#### White matter integrity: DTI measurements

The analysis of white matter integrity demonstrated that, overall, white matter near the lesion was not disproportionately compromised in terms of fiber count and fractional anisotropy.

DTI fiber count. A comparison of the number of corticospinal tract fibers between the LH group and control group revealed that LH patients had a significantly overall lower fiber count in the left hemisphere (LH group: *M* = 181.43; *SD* = 94.58; controls: *M* = 262.6, *SD* = 96.45) with respect to controls (*t*(27) = −2.29, p = 0.03, *d* = 0.85). A proportion was calculated (i.e., fiber count (LH)**/**fiber count (RH)) to elucidate whether or not connective integrity was disproportionately compromised in the left hemisphere. However, this proportion did not differ between groups (LH group: *M* = 0.93; *SD* = 0.58; controls: *M* = 1.07, *SD* = 0.48; *t*(27) = −0.72, p = 0.48).

The same analysis was performed for RH patients compared to controls. Unlike LH patients, there was no significant difference in neither the overall fiber count in the right hemisphere (RH group: *M* = 272.5; *SD* = 82.19; controls: *M* = 277.2; *SD* = 132.1; *t*(14.97) = −0.1, p = 0.92) nor the proportion (i.e., fiber count (RH)**/**fiber count (LH)) between hemispheres (RH group: *M* = 1.22; *SD* = 0.78; controls: *M* = 1.11; *SD* = 0.48; *t*(6.58) = 0.32, p = 0.76).

DTI fractional anisotropy (FA). A comparison of white matter integrity revealed no significant difference between LH patients’ FA values in the left hemisphere (LH group: *M* = 0.55; *SD* = 0.016; controls: *M* = 0.57, *SD* = 0.026) and that of controls controls (*t*(27) = −1.96, p = 0.063, *d* = 0.85). A proportion was calculated (i.e., FA (LH)**/**FA (RH)), which also revealed no significant difference (LH group: *M* = 1.03; *SD* = 0.06; controls: *M* = 1.03, *SD* = 0.05; *t*(27) = 0.09, p = 0.93).

**Figure 3:**
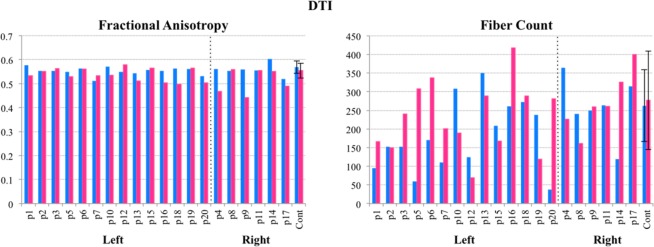
Plot of the Fractional Anisotropy (FA) and total fiber counts from the DTI analysis. Patients are divided by lesion hemisphere (Left or Right), with a dashed line indicating this division. Legend refers to the hemisphere of analysis.

The same analysis was performed for RH patients compared to controls. There was no difference in white matter integrity neither in the right hemisphere (RH group: *M* = 0.51; *SD* = 0.05; controls: *M* = 0.55; *SD* = 0.03; *t*(14.97) = −0.1, p = 0.92) nor in the proportion (i.e., FA (RH)/FA (LH)) between hemispheres (RH group: *M* = 0.91; *SD* = 0.09; controls: *M* = 0.97; *SD* = 0.04; *t*(9.61) = −0.17, p = 0.87). For a plot of patient performance compared to controls for both FA and fiber counts, see Fig. 3.

### Neuropsychological measures

An assessment of all experimental task measures is presented in Table 3. Overall, patients demonstrated significant impairment in performing the mental rotation of hands task and the

**Table 3:** Patients’ performance on experimental task performance.

P_ID	Les_Hem	FPIQ_Kin	FPIQ_Pos	FPIQ_Act	FPIQ_Obj	FPIQ_Tot	MR_ Hand	MR_ Feet	Gram_ Face	Gram_ Foot	Gram_Hand	Gram_ Neut	KD	BADA_V
1	LH	**5**	6	9	8	**28**	**24**	np	17	16	18	45	51	28
2	LH	6	8	9	10	33	26	**22**	18	18	17	47	41	28
3	LH	9	9	10	9	37	np	np	np	np	np	np	np	28
5	LH	8	6	9	8	31	**14**	np	17	17	18	47	38	**25**
6	LH	7	6	9	10	32	31	27	18	18	18	46	37	28
7	LH	6	**4**	**6**	9	**25**	**21**	np	17	15	15	41	**20**	**25**
10	LH	**5**	8	**5**	9	**27**	**18**	**14**	**8**	**9**	12	**22**	36	28
12	LH	**5**	7	10	8	30	**34**	25	16	17	17	45	45	28
13	LH	8	8	10	10	36	27	29	18	18	18	47	41	27
15	LH	7	6	10	10	33	29	25	12	14	13	33	38	28
16	LH	7	8	8	8	31	30	np	18	18	17	45	42	27
18	LH	7	7	**6**	9	**29**	**13**	**12**	11	11	12	34	36	26
19	LH	7	**4**	9	10	30	**16**	**22**	15	13	17	39	40	28
20	LH	**10**	9	9	10	38	33	30	18	17	18	45	46	28
4	RH	8	6	10	10	34	26	**16**	np	np	np	np	44	28
8	RH	9	6	10	10	35	29	np	np	np	np	np	44	28
9	RH	**5**	7	9	9	30	30	28	np	np	np	np	47	28
11	RH	6	9	8	9	32	**25**	**20**	np	np	np	np	32	28
14	RH	8	8	9	9	34	**14**	np	np	np	np	np	43	28
17	RH	8	9	9	10	36	31	28	np	np	np	np	41	28
Cont__M	—	*7.64*	*7.93*	*9.21*	*9.43*	*34.21*	*29.33*	*28.3*	15	15	*15.7*	*39.9*	*41.47*	—
Cont_SD	—	*1.01*	*1.94*	*0.89*	*0.94*	*2.75*	*2.3*	*2.79*	*3.5*	*3.2*	*2.5*	*8.13*	*6.4*	—

Performance that significantly differed from controls, as assessed by Z-score comparison, is presented in bold face.

Cont_M = control group mean; Cont_SD = control group standard deviation;

Les_Hem = Lesion Hemisphere; BADA_V = B.A.D.A. verb naming; FPIQ = Florida Praxis Imagery Questionnaire; Kin = kinesthetic subtest; Act = action subtest; Obj = object subtest; Tot = total score; KD = kissing and dancing test; MR = mental rotation; Gram = Grammar task; Face = face verbs; Foot = foot verbs; Hand = hand verbs; Neut = neutral verbs.

Florida Praxis Imagery Questionnaire; by contrast, they performed well on the Kissing and Dancing Test, on the grammar task and on naming verbs.

#### Mental rotation (MR)

Patients overall demonstrated the grossest deficits in performance on the mental rotation of hands task. Of the 19 patients that performed the task, 7 out of 13 LH patients and 2 out of 6 RH patients demonstrated performance outside of the normal range (average score: 0.52; maximum: *Z-*score = −1.87, p = 0.03) relative to controls (*M* = 0.86; *SD* = 0.07) irrespective of tumor site.

Next, we looked at the effect of the presence of a tumor in the hand region on performance deficits on hand MR. Of the 8 LH patients presenting with tumor to the hand region, 5 LH patients demonstrated significant reductions in MR performance (average score: 0.53; maximum: *Z*-score = −2.31. Furthermore, Pearson correlation revealed a significantly positive correlation between mental rotation performance and MEP amplitude (N = 5; r = 0.96, p = 0.008), meaning that performance on mental rotation was worse for lower MEP amplitudes, but that performance increased with increasing amplitude. This finding indicates a possible contribution of motor cortical regions to the ability to mentally rotate hands. To ensure that the hand-region ROI size did not contribute to the strength of MEP activations, a partial correlation was performed controlling for the number of voxels active when performing right hand movements in the localizer task. Importantly, even after considering this factor, a correlation between MR performance and MEP amplitudes was still present (N = 5; r = 0.99, p = 0.01).

On the mental rotation of feet task, 4 out of 9 LH patients and 2 out of 4 RH patients performed significantly worse (maximum: *Z* score = −2.26, p = 0.01) compared to control group performance (*M* = 0.83; *SD* = 0.08) irrespective of tumor site. Of the 4 LH patients demonstrating deficit, 3 (P2, P4, and P10) had a tumor closest to the foot region (average score: 0.47; maximum: *Z* score = −2.26, p = 0.01).

#### Florida praxis imagery questionnaire (FPIQ)

Four out of 20 patients demonstrated significant impairment on the *kinematics* subtest of the FPIQ, with 3 being LH patients (P1, P10, and P12; accuracy = 0.5 each; *Z-*score = −2.62 each; p = 0.004) and 1 (P9; accuracy = 0.5; *Z*-score = −2.62; p = 0.004) being RH, compared to control group performance (*M* = 0.76; *SD* = 0.1). Of the 4 LH patients, P1 showed an overall cumulative score deficit (accuracy = 0.7; *Z-*score = −2.26; p = 0.01) compared to control group performance (*M* = 0.86; *SD* = 0.07). P10 also demonstrated a reduction in performance on the *action* subtest (accuracy = 0.5; *Z-*score = −4.72; p < 0.001) compared to controls (*M* = 0.92; *SD* = 0.09) in addition to a cumulative score deficit (accuracy = 0.68; *Z-*score = −2.62; p = 0.004).

P18 (LH) also demonstrated impaired performance on the *action* subtest (accuracy = 0.6; *Z-*score = −3.6; p < 0.001) in addition to a cumulative performance that was significantly worse than controls (accuracy = 0.73; *Z-*score = −1.9; p = 0.023). P7 (LH) also demonstrated impaired performance on the *position* (accuracy = 0.4; *Z-*score = −2.02; p = 0.02; controls: *M* = 0.79; *SD* = 0.19) and *action* subtests (accuracy = 0.6; *Z-*score = −3.6; p < 0.001), in addition to a significant reduction in cumulative performance (accuracy = 0.63; *Z-*score = −3.35; p < 0.001). Finally, P19 (LH) also demonstrated impairment on the *action* subtest (accuracy = 0.4; *Z-*score = −2.02; p < 0.022).

A comparison of the total performance score between LH patients and the control group revealed a significant difference between groups [F(1, 26) = 5.01, p = 0.03, *d* = 0.85], with LH patients demonstrating an overall lower score than that of controls (LH patients: *M* = 31.43, *SD* = 3.76; controls: *M* = 34.21, *SD* = 2.75). We again considered a relationship between MEP amplitudes of the upper limb motor region and total FPIQ performance, but there was no significant correlation.

#### Kissing and dancing test (KDT)

Patients performed well on this task with the exception of P7 (LH) who demonstrated a deficit in performance (accuracy = 0.38; *Z-*score = 3.35; p < 0.001) relative to the control group (*M* = 0.8; *SD* = 0.12).

#### Grammar task

When considering any of the subtests of the grammar task, only LH patient P10 performed significantly worse than controls on the face (accuracy = 0.44; *Z-*score = −2; p < 0.023; controls: *M* = 0.8; *SD* = 0.19), foot (accuracy = 0.5; *Z-*score = −1.88; p < 0.03; controls: *M* = 0.8; *SD* = 0.18), and neutral (accuracy = 0.47; *Z-*score = −2.2; p < 0.01; controls: *M* = 0.85; *SD* = 0.18) verb subtests.

#### BADA verbs

Only two of the 14 LH patients, P5 and P7, performed slightly below (25/30) the cutoff (26/28). P7 had a slight generalized difficulty in naming nouns (27/30) with a (28/30) cutoff. None of the RH patients performed the task below the cutoff.

## Discussion

The primary aim of the current study was to investigate the involvement of sensorimotor regions in tasks that require motor simulation and lexical semantic processing utilizing a variety of neurophysiological measures. To this end, we tested 20 neurosurgical patients with right or left focal gliomas in somatotopic regions of the pre-and post-central gyrus. Our results indicated that left-hemisphere lesion patients performed significantly worse than controls in tasks engaging motor imagery. This pattern of performance was most consistent in the mental rotation of hand task and, to a lesser extent, feet task. This finding was corroborated by a modulation in the strength of MEP activations, which correlated with mental rotation performance.

When participants are presented with pictures of whole body or body parts and are asked to assess the handedness of the arm depicted, they implicitly compare it with a representation of their own arm, mentally rotating their own limb towards the orientation of the stimulus^62–64^ in order to match an egocentric map coding body position (i.e. body schema)^65^. In our analysis, statistical analysis revealed a significant effect of the presence of lesion in the somatotopic hand region on task performance. Unsurprisingly, this effect was observed for left-hemisphere tumor patients. Left-lateralization of motor imagery processing has been reported in a number of studies^27,66,67^. Furthermore, it has been hypothesized that two independent mechanisms differentially engage mental rotation based on stimulus type, hands versus objects, with rotation of the former implicitly triggering object-based spatial transformations that, in turn, automatically activate motor simulation^68,69^. While alternative interpretations have supported a strategy-driven response to successful performance of mental rotation^32,70,71^, it would appear that in our study, in the absence of instruction, participants automatically adopted a frame of reference (i.e., first-person perspective) that depends on sensorimotor imagery for successful task performance. It is unlikely that this automatic adoption of “strategy” can be explained by top-down processing to meet contextual task demands^32^, given that our patients possessed the cognitive capacity to employ such a mechanism, demonstrating general intact neuropsychological performance and no disproportionately-compromised connective integrity between hemispheres as indexed by DTI^72^. Therefore, it is most likely that body parts present a special type of stimulus that automatically engages sensorimotor imagery and activation^73^. A significant correlation was observed between mental rotation hand task performance in left hemisphere tumor patients and MEP amplitude in the upper limb motor region, furthering this claim. Similar relationships between MEP amplitudes and both mental rotation performance^74^ in addition to action word processing^13,16^ have been cited in the literature of healthy subjects.

A second evidence of impaired motor simulation ability was found when patients performed the Florida Praxis Imagery Questionnaire (FPIQ). We observed that on the FPIQ, a task that taps motor imagery ability, a number of patients performed significantly outside the range of controls across subtasks, with the most pronounced deficit observed in the kinesthetic subtest. Interestingly, while a smaller subset of patients demonstrated deviant performance on position and action subtests, all patients were within the normal range for the object subtest. The kinesthetic subtest, and arguably to a lesser extent, the action and position subtests, necessitate imagery from a first-person perspective, which purportedly dissociates from the kind of visual imagery that may have been triggered by the object subtask^75^. Moreover, although it is important to bear in mind that a greater number of left hemisphere than right hemisphere patients were tested, the majority of patients displaying deviant performance were left tumor patients. When comparing the total performance score between left tumor patients and controls, results indicated a significant reduction in performance in the patient group. Several studies have demonstrated a left lateralization effect in tasks involving action- versus non-action related sentence processing through the use of dynamic causal modeling^76^ and facilitation of mental imagery of action verbs through the use of TMS^77^.

By contrast, we found that tasks requiring the lexico-semantic processing of action related words were not compromised by lesion involving the sensorimotor area. Patients had an intact conceptual knowledge of the semantic relatedness of actions, performing well on the Kissing and Dancing Test^78^. This task has been found to be impaired in patients who have a deterioration of the representation of actions in language and semantics^79,80^. In addition, our patients performed normally on the verb-naming task. Our results are consistent with a previous study from our group indicating that neurosurgical patients’ ability to perform an action verb-naming task was not related to a damaged primary motor cortex; in addition, we had observed no significant changes in functional coupling between the left primary motor cortex and functional nodes of the linguistic network^81^. Rather, the naming of actions and action verb has been found to correlate with lesions in the parietal areas and in the posterior temporal cortex^82^ in addition to damage to the left middle and superior frontal lobe, the rolandic operculum, and the left inferior parietal lobule^83^.

Lastly, all but one patient (P10, who demonstrated a more ubiquitous task deficit) demonstrated normal performance across all verb categories on the grammar task. This finding is somewhat at odds with the hypothesis that verb processing necessitates sensorimotor activations of somatotopic body part representation. Papeo and colleagues^59^ found that TMS-induced MEPs in a relevant motor area (e.g., foot) increased for first-person action verbs but not for third-person action verbs, leading them to conclude that perceiving self as agent is critical for motor simulation. Embodied accounts have claimed that internal simulation of a described action is automatically perceived in an egocentric first-person perspective in the absence of explicit instruction^84–86^. However, our results indicate that the involvement of sensorimotor areas by first person verbs may not be as automatic as previously conceived.

One major limitation of our study is the small sample size; however, this is a natural limitation given by our population of study. Lesion patients are a rare population and difficult to recruit. However, given the precision of cortical localization that lesion patients offer, we feel that this did not significantly impact the reliability of our results. Notably, the data were collected from pre-surgical patients; it would have been interesting to also test the same patients post-operatively in order to compare potential changes in task performance such as spontaneous recovery of motor imagery abilities. Furthermore, important insight into the role of sensorimotor cortices in successful task execution could also be provided by testing patients intra-operatively, whereby immediate changes in motor abilities can be directly assessed as online feedback is provided in order to guide surgical mapping^87^. One final point of note is that we currently only analyzed accuracy data. It would have been interesting to have also considered reaction time data in order to measure chronometry of action simulation.

Taken together, our cognitive neuropsychological evaluations of neurosurgical patients with focal lesions allowed us to directly test the predictions of the embodied view of conceptual processing, namely the necessary involvement of the sensorimotor cortex in action verbs processing. While previous studies in both healthy and patient studies have reported somatotopic activation when processing action verbs associated with specific body parts^9,10,20,88^, support from patient studies has been limited by the diffusivity of the lesion that has extended beyond primary motor and sensorimotor cortices^89^. Our results showed that only some tasks known to induce motor simulation- mental rotation of hands task^73^ and the subtest of the FPIQ requiring kinesthetic imagery- were impaired following a lesion in sensorimotor areas whereas other tasks postulated by full embodied theories to require embodiment (e.g., lexico-semantic processing), were performed well by the same patients. We also showed that while patients naturally demonstrated voxel counts outside of the range of normality, this finding could not account for the correlation between MEPs and mental rotation hand task performance witnessed in left hemisphere tumor patients. Thus, our results do not support the view that sensorimotor regions are necessary to lexico-semantic processing as full embodied theories would predict.

## Supplementary information

Supplementary information.

## Data Availability

All data presented in the manuscript is available upon request.
