# Supplementary material for: Neurosurgical lesions to sensorimotor cortex do not impair action verb processing
Source: Sci Rep. 2020 Jan 16;10:523. doi: 10.1038/s41598-019-57361-3 (PMC6965077; doi:10.1038/s41598-019-57361-3)
Supplement: Supplementary file 1 — Supplementary information. [file 41598_2019_57361_MOESM1_ESM.docx]

**Neurosurgical lesions to sensorimotor cortex do not impair action verb processing**

Georgette Argiris^a*^, Riccardo Budai^b^, Marta Maieron^c^, Tamara Ius^d^, Miran Skrap^d^ & Barbara Tomasino^a^

*^a^ Scientific Institute IRCCS “Eugenio Medea”, Polo FVG, San Vito al Tagliamento (PN), Italy*

*^b^ Unità Operativa di Neurologia,* Azienda *Sanitaria* Universitaria *Integrata* S. *Maria della Misericordia, Udine, Italy*

*^c^ Fisica Medica,* Azienda *Sanitaria* Universitaria *Integrata* S. *Maria della Misericordia, Udine, Italy*

*^d^ Unità Operativa di Neurochirurgia,* Azienda *Sanitaria* Universitaria *Integrata* S. *Maria della Misericordia, Udine, Italy*

**8. Supplementary Material**

*Experimental Tasks*

*S1.1 Florida Praxis Imagery Questionnaire (FPIQ)*

General motor imagery ability was assessed using an adapted Italian computerized version of the Florida Praxis Imagery Questionnaire (FPIQ)^53^. Participants were presented with 10 out of the original 12 action scenarios (e.g., imagine you are trying to unlock a door), one at a time, and asked four types of forced choice questions that tapped into different aspects of learned skilled movement (for a total of 40 items). Two action scenarios were not used because the objects involved (i.e., ice pick and nail file) were not known to all participants. These four questions comprised four subscales relating to *kinesthetic* *imagery* (i.e., movement of the joints used to perform the action), *position imagery* (i.e., spatial location of the hands when performing the action), *action imagery* (i.e., motion of the limb when performing the action) and *object* *imagery* (i.e., information about the object upon which the action is performed) imagery. An example of each subscale is listed below, for the instructions *"Imagine you are using a handsaw..."*

a.) Kinesthetic imagery: Which joint moves more, your shoulder or your wrist?

b.) Position imagery: Which is lower, your index finger or your pinky?

c.) Action imagery: Does your hand move up and down or front to back?

d.) Object imagery: Is a handsaw wider at the top or at the handle?

Participants were presented with each of the four subsets and instructed the following: You are asked to imagine performing the following actions. After the presentation of each action, you will be asked a question to which you must respond by pressing 1 or 2 on the screen using the mouse.

Scenarios were presented one at a time in size 50 font. For each scenario, a fixation cross (1000ms) was followed by the presentation of the action (e.g., imagine you are trying *to saw* a tree) and a question belonging to one of the four subscales with two possible answers (e.g., *kinesthetic:* Do you move more your 1.) shoulder or 2.) wrist?). The question remained on the screen until a response was provided. Responses were coded as correct (1 point) or incorrect (0 points).

*S1.2. Mental Rotation (MR) of hands task*

Mental imagery ability was assessed by the presentation of a mental rotation task in which participants were presented with rotated images of hands and feet. Two sets of color photographs (resolution: 574 x 596 pixels), one set comprising 34 open hands and the other 34 feet (all digits fully extended, 50% right and 50% left) were used as stimuli. In each of the two sets, *view* (palm up or palm down for hands/sole up or sole down for feet) and *orientation* (rotated in increments of 45°; range: 0° to 315°) were manipulated.

The task was divided into two parts, with participants first viewing hands images and then foot images. Participants were instructed the following: You will be presented with images of hands (or feet). You must decide if the hand (or foot) is right or left. If right, please press 1 on the screen and if left, please press 2 on the screen, using the mouse.

Each trial consisted of the presentation of a fixation cross (1000ms), followed by the presentation of the image (3200ms), necessitating a response within that time. If no response was made, the display proceeded to the next trial and a score of 0 was given. Both reaction times and correct response were recorded.

*S1.3. Kissing and Dancing Test (KDT)*

To evaluate conceptual knowledge of actions, we presented participants with the written verb subtest of the Kissing and Dancing Test (KDT)^75^ adapted for Italian speakers. The test consisted of 52 triplets of words representing verbs that employed a match-to-sample scheme. In each triplet, a probe verb was presented at the top part of the screen and two comparison verbs in the lower part of the screen.

Participants were instructed the following: Read the following verbs. Decide which of the two verbs presented at the bottom is more semantically related to the verb at the top by pressing 1 or 2 on the screen, using the mouse.

Written verbs were presented in size 50 font. Each trial consisted of the presentation of a fixation cross (1000 ms), followed by the presentation of the verb triplets (3600 ms), necessitating a response within that time. If no response was made, the display proceeded to the next trial and a score of 0 was given.

*S1.4. Grammar task*

Previous research supporting embodied views has shown that transcranial magnetic stimulation (TMS)-induced MEPs do reflect motor simulation during action-verb processing, but that this effect is limited to first-person, and not third-person, verbs^57^. Thus, in order to assess lexical processing at the level of grammar, subjects were presented with 101 Italian action verbs relating to *hand* [H]- (N= 18 e.g., “to grasp”), *face* [FA]- (N= 18, e.g., “to chew”), and *foot* [FO]- (N= 18, e.g., “to kick”) movements in addition to neutral verbs [N] (N= 47 e.g., “to suffer”); ~25% were presented in the first-person singular, ~50% in the third-person singular, and ~25% in the third-person plural. Singular and plural forms were used in order to increase task difficulty and maintain attention. These stimuli were a chosen subset of those previously used by Tomasino et and colleagues^55^, which were originally taken from the Italian corpus of Laudanna and colleagues^56^. The stimuli were chosen based on having received 95% agreement across 20 healthy adult raters in judging the stimuli as belonging to one of the four chosen categories. Stimuli were matched for both frequency and length across categories (*P* > .05).

Participants were instructed the following: Silently read the following verbs and decide whether or not each verb is in the third-person singular form, by pressing 2 if yes and 1 if no, on the screen by using the mouse. Trials were presented in a randomized order. Performance was measured as the number of correct responses.

*S1.5 Verb naming task*

Patients’ ability to name verbs was tested by using the verb oral naming task from the Batteria per L'Analisi dei Deficit Afasici (BADA)^51^. The verb naming task includes 28 items, half being high-frequency words and the remaining half being low-frequency words. Participants were asked to carefully look at the stimulus pictures and name the corresponding verb. Pictures were presented individually on an A4 sheet of paper. For each picture, the experimenter asked: “What is he/she/it doing?”. Answers were recorded and transcribed by the experimenter for later analysis.

*S2. Table 1*

| **P_ID** | **Raven*** | **Digit_F*** | **Digit_B*** | **B_Apr** | **I_Apr** | **Token** | **BADA_N** | **FAS*** | **Corsi_F*** | **Corsi_B*** | **C_Apr** | **CDT** | **B_Att** | **B_Let** | **B_ Line** | **Neg** | **TMT_A** | **TMT_B** |
| --- | --- | --- | --- | --- | --- | --- | --- | --- | --- | --- | --- | --- | --- | --- | --- | --- | --- | --- |
| 1 | 31.5 | 6.02 | 3.97 | 20 | 72 | 36 | **27** | 37 | np | np | np | np | np | np | np | np | np | np |
| 2 | 28 | 5.68 | 3.64 | 20 | 72 | 35 | 30 | 32 | np | np | np | np | np | np | np | np | np | np |
| 3 | 30 | 5.55 | 3.31 | 20 | 72 | 36 | 28 | **6** | np | np | np | np | np | np | np | np | np | np |
| 5 | 31 | 5.13 | 4.08 | 20 | 64 | 34 | 30 | **13** | np | np | np | np | np | np | np | np | np | np |
| 6 | 31 | 5.3 | 4.21 | 20 | 72 | 36 | 30 | 37 | np | np | np | np | np | np | np | np | np | np |
| 7 | 27.5 | 7.02 | 2.97 | 20 | 66 | 31 | **27** | 22 | np | np | np | np | np | np | np | np | np | np |
| 10 | 29 | 7.13 | 3.08 | 20 | 72 | 34 | 30 | 31 | np | np | np | np | np | np | np | np | np | np |
| 12 | 26 | 5.68 | 4.64 | 20 | 72 | 36 | 30 | 58 | np | np | np | np | np | np | np | np | np | np |
| 13 | 29 | 5.47 | 3.37 | 20 | 72 | 35 | 30 | 29 | np | np | np | np | np | np | np | np | np | np |
| 15 | 32.5 | 7 | 5 | 20 | 71 | 36 | 28 | 45 | np | np | np | np | np | np | np | np | np | np |
| 16 | 30 | 5.55 | 3.52 | 20 | 70 | 36 | 30 | 48 | np | np | np | np | np | np | np | np | np | np |
| 18 | 34 | 5.04 | **2.1** | 19 | **43** | 32 | 29 | **10** | np | np | np | np | np | np | np | np | np | np |
| 19 | 34.5 | 6.04 | 4.1 | 20 | 72 | 36 | 30 | 31 | np | np | np | np | np | np | np | np | np | np |
| 20 | 31.5 | 6.75 | 3.71 | 20 | 70 | 36 | 30 | 36 | np | np | np | np | np | np | np | np | np | np |
| 4 | 30 | np | np | np | np | np | np | np | 4.86 | **3.48** | 13 | 10 | 54 | 40 | 9 | 40 | 33 | 71 |
| 8 | 27 | np | np | np | np | np | np | np | 5.32 | 5.31 | 14 | 10 | 54 | 39 | 9 | 40 | 49 | 149 |
| 9 | 30.5 | np | np | np | np | np | np | np | 5.37 | **3.39** | 14 | 10 | 54 | 39 | 9 | 40 | 33 | 102 |
| 11 | 29.5 | np | np | np | np | np | np | np | 4.44 | **3.24** | 14 | 10 | 54 | 40 | 9 | 40 | 31 | 111 |
| 14 | 29 | np | np | np | np | np | np | np | **3.54** | **3.39** | 12 | 10 | 54 | 40 | 9 | 40 | 31 | 100 |
| 17 | 32 | np | np | np | np | np | np | np | 5.92 | **3.57** | 14 | **7.5** | 54 | 40 | 9 | 40 | 16 | 37 |
| Cutoff | >=18 | >=4.26 | >=2.65 | >=11 | >=53 | >=25.26 | >=27 | >=16 | 3.75 | 3.75 | 8 | 8.8 | 51 | 34 | 7 | 35 | > 94 | > 283 |

***S2. Table 1.*** *LH and RH patients' neuropsychological screening*

*tasks normalized by age/education; values below the cutoff are bolded

Digit_F = Digit Span (Forward); Digit_B = Digit Span (Backward); B_Apr = Buccofacial Apraxia; I_Apr = Ideomotor Apraxia; BADA_N = B.A.D.A. noun naming; FAS = phonological fluency; Corsi_F = Corsi Block-tapping (Forward); Corsi_B = Corsi Block-tapping (Backward); C_Apri = constructional apraxia (figure drawing); CDT = clock-drawing test; B_att = BIT attention; B_Let =BIT letter cancellation; B_Line = BIT line bisection; Neg = BARRAGE (hemispheric neglect); BADA_V = B.A.D.A. verb naming

*S2. Table 2.*

| **ID** | **Les_Hem** | **Mouth_LH** | | | **Mouth_RH** | | | **Hand_LH** | | | **Hand_RH** | | | **Foot_LH** | | | **Foot_RH** | | |
| --- | --- | --- | --- | --- | --- | --- | --- | --- | --- | --- | --- | --- | --- | --- | --- | --- | --- | --- | --- |
|  |  | **x** | **y** | **z** | **x** | **y** | **z** | **x** | **y** | **z** | **x** | **y** | **z** | **x** | **y** | **z** | **x** | **y** | **z** |
| 1 | LH | ne | ne | ne | ne | ne | ne | -36 | -24 | 56 | 40 | -24 | 60 | -8 | -38 | 70 | 6 | -34 | 70 |
| 2 | LH | -48 | -12 | 40 | 50 | -10 | 32 | -34 | -24 | 50 | 30 | -20 | 52 | -2 | -34 | 70 | 8 | -38 | 72 |
| 3 | LH | -48 | -14 | 38 | 46 | -12 | 34 | -38 | -18 | 56 | 38 | -30 | **66** | -14 | -42 | 74 | **14** | -38 | 72 |
| 5 | LH | -44 | **-18** | 44 | 50 | -8 | 34 | -38 | -28 | 66 | 30 | -22 | 54 | -4 | -28 | 74 | 2 | -32 | 64 |
| 6 | LH | -46 | -14 | 34 | 54 | -8 | 38 | -34 | -22 | 60 | 34 | -24 | 60 | -4 | -30 | 72 | 6 | **-26** | 72 |
| 7 | LH | -50 | -12 | 38 | 54 | -10 | 44 | -36 | **-14** | 50 | 36 | -26 | 52 | -2 | **-26** | **60** | 2 | -34 | 60 |
| 10 | LH | ns | ns | ns | ns | ns | ns | -38 | -26 | 54 | 36 | -26 | 54 | -4 | -38 | **62** | 0 | -28 | **58** |
| 12 | LH | -50 | -8 | 38 | 50 | -10 | 40 | -38 | -22 | **46** | 38 | -16 | 50 | ne | ne | ne | ne | ne | ne |
| 13 | LH | -42 | -8 | 34 | 48 | -12 | 38 | -34 | -24 | 62 | 40 | -18 | 54 | -4 | -40 | 64 | 12 | **-42** | 76 |
| 15 | LH | ns | ns | ns | ns | ns | ns | **-32** | -28 | 66 | 40 | -22 | 60 | -6 | -40 | 72 | 2 | -32 | **56** |
| 16 | LH | -48 | -12 | **32** | 56 | -6 | 36 | -40 | -24 | 58 | 40 | -24 | 52 | -14 | -38 | 74 | **14** | -34 | 74 |
| 18 | LH | -54 | -10 | 42 | 56 | **-4** | 32 | -38 | -28 | 50 | 34 | **-32** | 56 | -8 | -32 | 72 | 12 | -30 | 70 |
| 19 | LH | -44 | -14 | 36 | 50 | -6 | 36 | -40 | -26 | 60 | 38 | -18 | 50 | -12 | -38 | 72 | 4 | -38 | 72 |
| 20 | LH | ns | ns | ns | 58 | -8 | 38 | **-32** | -26 | 52 | 38 | -24 | 52 | -8 | -38 | 68 | 2 | -30 | 60 |
| 21 | LH | -52 | -12 | 44 | 59 | -8 | 40 | **-32** | -28 | 66 | 40 | -22 | 60 | -14 | -40 | 72 | 0 | -28 | 64 |
| 23 | LH | ns | ns | ns | **42** | -14 | 40 | ns | ns | ns | 40 | -20 | 56 | -10 | -42 | 76 | 0 | -32 | 70 |
| 4 | RH | ns | ns | ns | ns | ns | ns | -36 | -28 | 48 | **26** | -30 | **68** | -10 | -38 | 68 | 6 | -38 | 76 |
| 8 | RH | **-56** | -12 | 44 | 56 | -6 | 36 | -36 | -26 | 60 | 40 | -22 | 60 | -8 | -42 | 74 | 4 | -38 | 68 |
| 9 | RH | -52 | -10 | 40 | ns | ns | ns | -38 | -22 | 60 | ns | ns | ns | -2 | -32 | 70 | 2 | -40 | 68 |
| 11 | RH | -46 | -16 | 46 | 50 | -10 | **44** | -36 | **-14** | 50 | 40 | -24 | 58 | -12 | **-50** | 72 | 6 | -38 | 72 |
| 14 | RH | -46 | -14 | 38 | 46 | **-16** | 38 | ne | ne | ne | ne | ne | ne | ne | ne | ne | ne | ne | ne |
| 17 | RH | ns | ns | ns | ns | ns | ns | -36 | -20 | 58 | 44 | -16 | 54 | -8 | -32 | 70 | 8 | -32 | 70 |
| **min** |  | -56 | -18 | 32 | 42 | -16 | 32 | -40 | -28 | 46 | 26 | -32 | 50 | -14 | -50 | 60 | 0 | -42 | 56 |
| **max** |  | -42 | -8 | 46 | 59 | -4 | 44 | -32 | -14 | 66 | 44 | -16 | 68 | -2 | -26 | 76 | 14 | -26 | 76 |
| **mean** |  | -48,14 | -12,4 | 39,2 | 51,56 | -9,25 | 37,5 | -36,1 | -23,6 | 56,4 | 37,1 | -23 | 56,4 | -7,7 | -36,9 | 70,3 | 5,5 | -34,1 | 68,2 |
| **sd** |  | 3,95 | 2,74 | 4,19 | 4,80 | 3,17 | 3,61 | 2,46 | 4,33 | 6,21 | 4,37 | 4,42 | 5,01 | 4,11 | 5,67 | 4,16 | 4,58 | 4,47 | 5,90 |
| ***Ctrls*** |  | ***-50*** | ***-12*** | ***38*** | ***50*** | ***-12*** | ***36*** | ***-36*** | ***-26*** | ***52*** | ***36*** | ***-24*** | ***52*** | ne | ne | ne | ne | ne | ne |

***S2. Table 2.*** *Peak coordinates of hand, foot, and mouth regions for left (LH) and right (RH) hemispheres. At bottom, descriptive statistics for each coordinate plane (i.e., minimum, maximum, mean and standard deviations) are presented in addition to the coordinates of the mean peak activations for healthy controls.*

Performance that significantly differed from controls, as assessed by Z-score comparison, is presented in bold face.

Les_Hem = Lesion Hemisphere; LH = left hemisphere; RH = right hemisphere;

*ns =* not significant activation; *ne =* not executed; *ctrls* = data from controls subjects
